# Role of miR-221/222 in Tumor Development and the Underlying Mechanism

**DOI:** 10.1155/2019/7252013

**Published:** 2019-12-24

**Authors:** Qian Song, Quanlin An, Bing Niu, Xiaoling Lu, Ning Zhang, Xin Cao

**Affiliations:** ^1^Zhongshan Hospital, Institute of Clinical Science, Shanghai Medical College, Fudan University, Shanghai 200032, China; ^2^School of Life Sciences, Shanghai University, Shanghai 200444, China; ^3^College of Basic Medical Sciences, Department of Biochemistry and Molecular Biology, Second Military Medical University, Shanghai 200433, China; ^4^Department of Hepatic Surgery, Fudan University Shanghai Cancer Center, Shanghai 200032, China

## Abstract

MicroRNA-221/222 (miRNA-221/222, miR-221/222) is a noncoding microRNA which is widely distributed in eukaryotic organisms and deeply involved in the posttranscriptional regulation of gene expressions. According to recent studies, abnormal expressions of miR-221/222 are closely related to the occurrence and development of various kinds of malignant tumors. The role of miR-221/222 in tumor development and their potential molecular mechanism in various cancers, including liver cancer, colorectal cancer, cervical cancer, ovarian cancer, and endometrial carcinoma, are summarized and reviewed in this paper. Moreover, the potential translational biomarker role of abnormal miR-221/222 level in tumor or blood circulation for tumor diagnosis is also discussed.

## 1. Introduction

MicroRNAs (miRNAs) are short, endogenous, noncoding RNAs with a length of 18–25 nucleotides. In the human genome, several thousands of miRNAs are encoded, which regulate more than 30% of the genes, thereby participating in the regulation of almost all cellular functions, such as cell differentiation, proliferation, growth, migration, and apoptosis. As illustrated, microRNA-221/222 (miRNA-221/222, miR-221/222) is a noncoding microRNA which is widely distributed in eukaryotic organisms and deeply involved in the posttranscriptional regulation of gene expressions [1–4].

**Figure 1:**
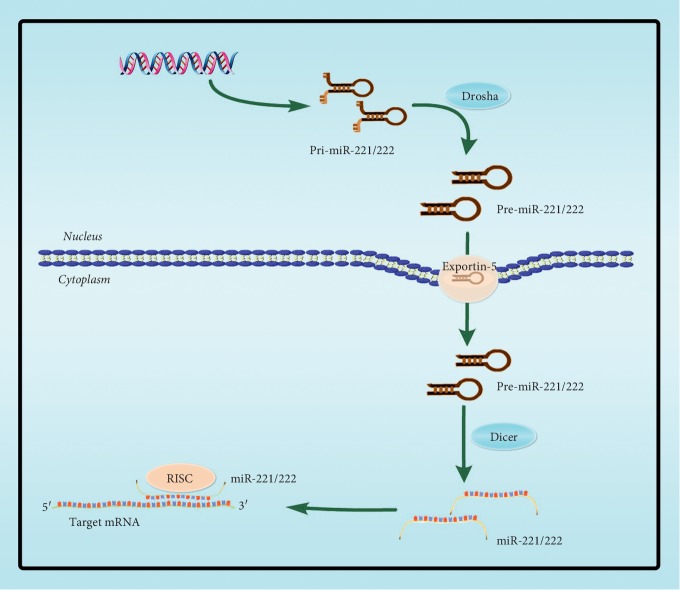
Biogenesis process of miR-221/222. The gene of miR-221/222 is transcribed into primary miR-221/222 (pri-miR-221/222) by RNA polymerase II in nucleus and then processed to a stem-loop precursor miR-221/222 (pre-miR-221/222) by the nuclear RNase Drosha in the nucleus. Pre-miR-221/222 is transported from the nucleus to the cytoplasm by the exportin-5 transporter, in which the endoribonuclease Dicer cleaves it into a double-stranded miRNA, one of which binds to the RNA-induced silencing complex (RISC).

Hsa-miR-221 and hsa-miR-222 are encoded tandemly in chromosome Xp11.3, which are highly homologous miRNAs sharing the same “seed sequence.” The present study investigated the role of miR-221/222, which plays an important regulatory role in the development and progression of tumors, both in promoting or suppressing cancers [5]. Since there are different subtypes like miR-221/222 3p or 5p, whose heterogeneity may perform different biological functions, their new progresses are also reviewed in this paper. The biogenesis of miR-221/222 is similar to other miRNAs' biogenesis, which is initially transcribed principally by either RNA polymerase II or RNA polymerase III as long primary transcriptions and is further processed by the nuclear RNase Drosha and cytoplasmic RNase Dicer to generate precursor miRNAs and mature miRNAs, respectively (Figure 1) [6]. In the cytoplasm, miRNAs recognize and bind to partially complementary sites in the 3′ untranslated region (3′UTR) of target mRNAs, resulting in either translational repression or target degradation [7].

Recently, accumulated investigations showed that the abnormal expression of miR-221/222 involved in various tumor initiation and progressions. As noted, abnormal miR-221/222 expression promotes cancer cell proliferation, invasion and metastasis, stemness promotion, cell survival, chemoresistance, or the relapse of cancer cells [5, 8]. In this review, the role and its research progress of potential mechanism of miR-221/222 in tumor development are summarized and discussed.

## 2. Role of miR-221/222 in Tumorigenesis and Progression

### 2.1. Upstream Regulation of miR-221/222

In recent years, studies have found that in miRNA biogenesis (Figure 1), the pri-miRNA hairpins by Drosha and Dicer could result in a change of the length at the 5′- or 3′-end of miRNA [9]. Thus, the isoform of miRNA is longer or shorter than the consensus miRNA length [10]. The heterogeneity at the 5′-end could result in “seed shifts,” thereby changing the expected pool of target genes [11], while the occurrence of 5′ variation is relatively rare. In contrast, the 3′ heterogeneity is discovered as more common situations [12–14]. As an essential endonuclease to produce mature miRNAs, more researchers suggested Dicer is related to the regulation of miR-222. Ueda et al. reported [15] that Dicer-disrupted cells exhibited upregulated intercellular cell adhesion molecule-1 (*ICAM-1*) expression and enhanced susceptibility to CTL-mediated cytolysis and directly demonstrated that miR-222 functionally targeted the 3′ UTR of *ICAM-1* mRNA according with luciferase reporter assays. After transfected with Dicer siRNA, or inhibitors of miR-222, the results of flow cytometric analyses and immunoblotting suggested the inhibition of Dicer or miR-222 led to an increase in *ICAM-1* expression in human malignant glioma cells and promoted their susceptibility to cytotoxic T-lymphocytes (CTLs). Zhang et al. suggested that adenosine deaminases acting on RNA(ADAR) 1 could promote the expression of miRNA-222 by complexing with Dicer and the expression of phosphatase-and-tensin (PTEN) protein was significantly reduced by miRNA-222 target *PTEN* gene's 3′UTR [16]. Chen et al. found that Dicer expression was significantly increased in gefitinib-resistant non-small-cell lung cancer (NSCLC) PC9/GR cell line compared with the gefitinib-sensitive NSCLC PC9 cell line, while the silenced Dicer induced sensitivity to gefitinib in NSCLC cells through the downregulation of miR-221/222 to increase the protein level of caspase-3 [17]. Cochrane et al. found that miR-221/222 was higher in estrogen receptor 1 (ESR1) negative breast cancer cell lines than positive breast cancer cell lines and miR-221/222 directly targets on *Dicer* and *ESR1* [18].

**Figure 2:**
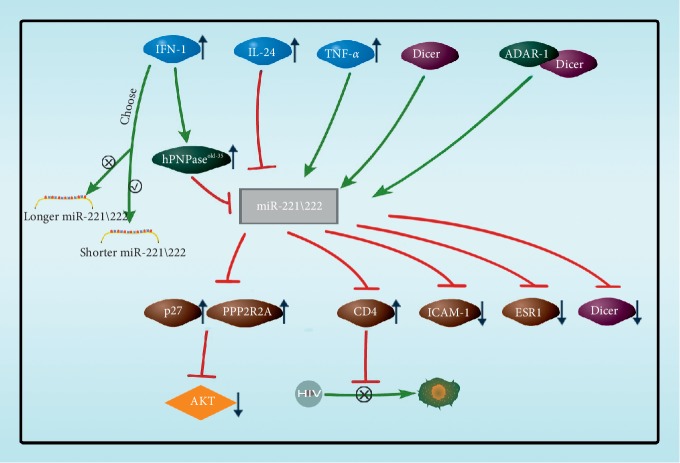
Cytokine regulations on miR-221/222. IFN-1 decreased the levels of longer miR-221/222 isoforms and increased the levels of shorter miR-221/222 isoforms. The longer miR-222 reduces cell fusion and promotes cell apoptosis by downregulating PI3K-AKT. miR-221/222 downregulated by IFN-1 and IL-24 treatment, and downregulation of miR-221/222 upregulated the target *p27* and protein phosphatase 2 regulatory subunit B alpha (*PPP2R2A*). But miR-221/222 was upregulated by TNF-*α* produced by macrophages and downregulation of miR-221/222 inhibits CD4 receptor expression in macrophages to inhibit HIV-1 entry into macrophages. Dicer generates mature miR-222, which suppresses *ICAM-1* expression on tumor cells. ADAR-1 complexing with Dicer can promote the expression of miR-222. miR-221/222 was higher in ESR1-negative breast cancer cell line and directly targets *Dicer* and *ESR1*.

Cytokines play key regulatory roles in the expression of miR-221/222 subtypes (Figure 2). Human polynucleotide phosphorylase (*hPNPaseold-35*), a highly evolutionarily conserved gene that catalyzes 3′-to-5′ phosphorolysis as well as 5′-to-3′ polymerization of RNA, acts as a trimer in the expression of miR-221/222 subtypes. Das et al. [19] reported that the overexpression of *hPNPaseold-35* by type-I IFN treatment resulted in robust and preferentially targeted downregulation of miRNA-221/222 with consequent upregulation of its suppressed target cyclin-dependent kinase inhibitor p27^Kip1^. Nejad et al. discovered that type-I IFN stimulation selectively decreases the levels of longer miR-221-3p and miR-222-3p isoforms, while sparing the shorter ones [20]. Yu et al. [21] analyzed the endogenous length variation across breast cancer tumor samples and observed the frequent occurrence of templated 3′ isomiRs of miRNA-221/222. In breast cancer cell line MCF10A, the longer miR-222 (extending 2–4 nucleotides at the 3′ end) reduced cell fusion and promotes cell apoptosis by downregulating PI3K-AKT, and the longer miR-222 displayed an increased proclivity for nuclear localization, which indicates that different subtypes of miRNA may have different functions. And when these subtypes are regulated, cellular activity is also affected. Panneerselvam et al. [22] found that IL-24 could decrease the levels of miR-222-3p and -5p, thus increasing the target PPP2R2A level of miR-222, inhibiting the activation of protein kinase B (PKB, AKT), and inhibiting the expression of AKT in lung cancer cells. Lodge reported [23] that the cytokine TNF-*α* produced by macrophages could enhance the expression of miR-221/222 and that miR-221/222 binds to *CD4* mRNA. It could also be found that cytokines not only regulate the expression of miR-221/222 but also selectively reduce the level of allogeneic cells in order to realize the regulation of cell activity.

### 2.2. Regulation of Competing Endogenous RNA on miR-221/222

The proposition of competing endogenous RNA (ceRNA) was based on a series studies on the “talk” between RNAs [24]. They present a hypothesis about ceRNA as that the reversed RNAs influence each other's levels by competing for a limited pool of miRNAs, which is essentially referred to “RNA ⟶ miRNA” and also a supplement to the traditional theory of “miRNA⟶RNA.” In the context of cancer, pseudogenes and long noncoding RNAs (IncRNAs) could act as potential tumor suppressors and oncogenes through their ceRNA function by binding miRNA response elements (MREs). Lu et al. used molecular network-based identification of ceRNA (MNIceRNA) to construct miRNA-mRNA-IncRNA networks in thyroid carcinoma, while the hsa-miR-221/222 acted as key driver RNAs during the process [25]. Liu et al. verified that the expression of IncRNA GAS5 was significantly lower in the HCT116 and SW480 cell lines compared with that in the normal NCM460 cell line, whereas the expression of miR-222-3p was significantly higher [26]. The dual-luciferase reporter assays, RNA immunoprecipitations (IP), and RNA pull-down assays revealed that miR-222-3p could specifically bind to PTEN and IncRNA GAS5 and suggested that IncRNA GAS5 could regulate PTEN through competitively binding to miR-222-3p.

### 2.3. miR-221/222's Targets and Molecular Modulation Pathways

*PTEN*, *TIMP3* (tissue inhibitor of metalloproteinases 3), *p27*, and *p57* are well-known tumor suppressor genes, but their expressions are often dysregulated in human cancer [27]. The research showed that both miR-221 and miR-222 directly targeted the 3′UTR of *p27* and *p57* mRNA and decreased the protein level of *p27* and *p57*, thus activating the AKT pathway [28]. On the other hand, miR-221 and miR-222 also targeted *PTEN* and to induce TRAIL tolerance and enhance cell migration [29]. PUMA is a member of Bcl-2 protein family and has a strong proapoptotic effect [30], while miR-221/222 could bind to 3′UTR of *PUMA* mRNA to inhibit the expression of *PUMA* and then resist normal apoptosis [31] (Table 1).

### 2.4. miR-221/222 Dysregulation in Human Cancer

The abnormal high expression of miR-221/222 is involved in various cancers (Table 1), which promotes the malignant proliferation, immune escape, invasion, and metastasis of tumor cells [94]. Through the retrieval and statistics of the databases (including PubMed and Web of Science), the oncogene upregulated with overexpression of miR-221/222, including glioblastoma [31], gastric cancer [32, 33], bladder cancer [35], hepatocellular carcinoma [38, 39, 51, 59], lung cancer [36, 37], liver cancer [29, 51], breast cancer [60–63], cervical cancer [64, 68, 95], ovarian cancer [66, 69, 70], endometrial carcinoma [74], melanoma [75, 76], pancreatic cancer [77], thyroid cancer [78, 79], multiple myeloma [80], chronic lymphocytic leukemia [79], oral carcinoma [82], retinoblastoma [83, 96], nasopharyngeal carcinoma [84], and prostate carcinoma [85], was summarized. However, the upregulation of miR-221/222, also referred to as tumor suppressor miRNA, targeting overexpress oncogenes, leads to tumor suppressions. For example, miR-221/222 cluster directly targeted Ecm29 and attenuated migration and invasion in PCa cells in prostate cancer [61]. miRNA-221/222 also targets on *kit*^+^ in erythroleukemic cells [86] and gastrointestinal stromal tumor pathogenesis [87], to inhibit cell proliferation and induce apoptosis. Functional analysis and luciferase reporter gene assays indicated that has-miR-222 inhibits oral tongue squamous cell carcinoma cell invasion by targeting on *matrix metalloproteinase 1* (*MMP1*) and manganese superoxide dismutase 2 (*SOD2*) mRNAs [88]. Furthermore, in HuH28 human cholangiocarcinoma cells' research, Okamoto and colleagues found miR-221 could target phosphoinositide-3-kinase, regulatory subunit 1 (*PIK3R1*), to inhibit HuH28 cell proliferation and conferred Gem sensitivity [89].

## 3. Role of miR-221/222 in Various Kinds of Cancers

### 3.1. Role of miR-221/222 in Liver Cancer

Hepatocellular carcinoma (HCC) is one of the most high-mortality malignant tumors [97]. Recently, Song et al. [98] published a review on effect of overexpression of miR-221/222 on liver cancer several years ago, focusing on the relationship between overexpression of miR-221 and HCC, while more details between miR-221 and -222 and HCC are reported in recent papers [38, 79, 90, 91, 99]. Several papers demonstrated that the miR-221/222 level is related to the tumor TNM stages [39, 100, 101], among which Fu et al. found miR-221 is mainly located in the plasma firstly [102]. Li et al. [103] found the miR-221/222 levels were higher in HCC patients, and the overall survival rate of the high miR-221 expression group was significantly lower than that of the low miR-221 expression group according to the analysis of 46 HCC patients and 20 healthy normal controls by RT-PCR. Sohn et al. [104] investigated the levels of serum exosomal miRNAs in HCC patients and compared them with the levels observed in chronic hepatitis B (CHB) or liver cirrhosis (LC) patients. As a result, the levels of serum exosomal miR-221 and miR-222 were significantly higher in patients with HCC than in patients with CHB or LC.

It has been demonstrated that PTEN, TIMP3, and p27/CDKN1B are identified as the targets of miR-221/222 in HCC samples [29, 51]; thereby, the G1/S transition is loss of control in HCC. Gramantieri and colleagues [41] used three prediction algorithms (miRanda, TargetScould, and PicTar) predicted and the luciferase reporter assay verified bone marrow failure (BMF), a proapoptotic BH3-only member of the Bcl-2 family, is the target of miR-221 [105]. Recent reports showed that miR-222 targeted on 3′UTR of the mRNA of B-cell lymphoma-2 binding component 3 (BBC3) in HepG2 cell line [48]. Wong et al. [39] suggested that the AKT signaling was the major pathway influenced by miR-222 and that PPP2R2A was a miR-222 target in silico, thereby enhancing HCC cell invasion and motility. Pineau et al. [51] observed miR-221 expression could distinguish malignant from adjacent cirrhotic tissues and identify DNA damage-inducible transcript 4 (DDIT4) as a direct target of miR-221. Ectopic overexpression of histone deacetylase 6 (HDAC6) causes JNK/c-Jun activation, leading to autophagic cell death in HCC cells [106]. Bae et al. [42] found that the miR-221 increased according to the Met/JNK-activated c-Jun and cytokine-induced NF-*κ*Bp65 transcription factor activation, while the miR-221 suppressed HDAC6 and thus promoted hepatocellular malignant transformation and proliferation. Zhang et al. [47] found alpha inhibiting activity polypeptide 3 (GNAI3) inhibits HCC cell migration and invasion, but GNAI3 is downregulated in HCC at the protein level but not at the mRNA level. They also identify miR-222 directly binds to the 3′UTR of GNAI3 and posttranscriptionally regulates GNAI3 expression. Epithelial-mesenchymal transition (EMT) increases the migratory capacity of epithelial cells and plays a key role in the metastasis of cancer cells [105]; some reports show that the miR-221 promotes EMT in HCC cell lines by targeting on adiponectin receptor 1 (AdipoR1) and cytokine signaling 3 (SOCS3), which implies that the JAK/STAT3 signaling pathway may be involved [44, 45]. Hepatitis B virus X (HBx) protein plays an important role in the development of hepatocellular carcinoma (HCC). Chen et al. [46] identified that miR-221 promotes HBV-related HCC cancer cell proliferation by directly targeting on estrogen receptor 1 (ER*α*).

**Figure 3:**
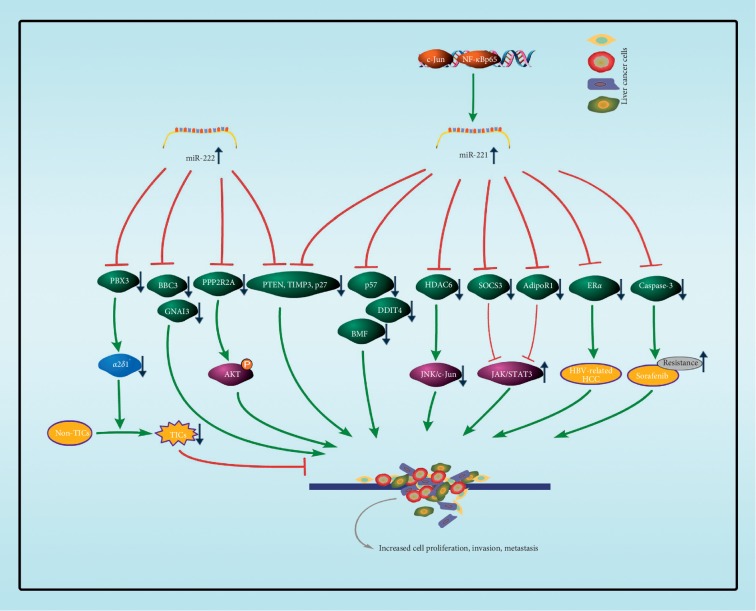
The main targets of miR-221/222 involved in liver cancer. miR-222 is a tumor suppressor by inhibiting *PBX3* expression, which activates the *α*2*δ*1 expression. *α*2*δ*1 is a surface marker that marks HCC tumor initiating cells (TICs). However, miR-221/222 is often used as an onco-miRNA in liver cancer cells. BBC3, GNAI3, and PPP2R2A have been identified as a target for miR-222 and p57. DDIT4, BMF, HDAC6, AdioR1, and ER*α* have been identified as a target for miR-221. PTEN, TIMP3, and p27/*CDKN1B* have been identified as a target for miR-221/222. The downregulation of these targets promotes liver cell proliferation, invasion, and metastasis.

miR-221/222 is not only acting as a biomarker in patients with HCC but also directly targets on key targets in liver cancer (Figure 3). Fornari et al. [43] demonstrated that caspase-3 is a direct target of miR-221 in HCC, further suggesting miR-221 inhibition as a therapeutic approach aimed to contrast resistance to sorafenib. Moreover, the roles of miRNAs are cell context-dependent. For example, Han et al. [50] confirmed that *α*2*δ*1 (encoded by the gene *CACNA2D1*) is a surface marker that marks HCC tumor-initiating cells (TICs), and they identified miR-222 as a tumor suppressor in HCC by controlling the stemness and tumorigenicity of *α*2*δ*1^+^ TICs by directly targeting on pre-B-cell leukemia homeobox 3 (PBX3), which activated the expression of *CACNA2D1*.

### 3.2. Role of miR-221/222 in Colorectal Cancer

Colorectal cancer (CRC) is one of the most common cancers and the fifth most common cause of cancer-related death world widely [97]. Several reports revealed miR-221/222 levels are elevated in human CRCs [58]. Early effective detection is critical for disease prevention; however, CRC is asymptomatic in the early stage and difficult to diagnose until advanced stages. Thus, there is a compelling need to identify molecular biomarkers for mass screening and early diagnosis of CRC. A report showed that miR-221 (*p* < 0.0001) was significantly higher in peripheral blood of 71 patients with colorectal cancer in comparison with 80 matched healthy control individuals [107]. Yau and colleagues evaluated the plasma miR-221 level [108] and the stool miR-221 level [109] and found that the plasma miR-221 had a high sensitivity (86%) but poor specificity (41%) and an AUC of only 0.61. In contrast, the stool miR-221 had an AUC of 0.73, a sensitivity rate of 62%, and a specificity rate of 74%; hence, the detection of miR-221 in stool is a better way. There are some reports that studied the changes of miR-221/222 in circulating tumor cells (CTCs) and miRNAs from CTCs during CRC treatment and found the changes in counts reflected the disease progression and/or response to chemotherapy; however, the miRNA levels (including miR-221/222) from CTCs showed transient expression and did not correlate with CTC counts [110].

The oncogene *KRAS* induces increased expression of miR-221/222 in human CRC cell lines [111]. It has been demonstrated that p27/*CDKN1B*, p57/*CDKN1C*, and PTEN have been identified as targets of miR-221/222 in CRC cells [49, 53, 54, 57]. Liao et al. [52] demonstrated that miR-221 promotes the cell proliferation of CRC via the inhibition of autophagy and targeted tumor protein 53-induced nuclear protein 1 (TP53INP1). Liu et al. [58] proposed a miR-221/222-NF-*κ*B-STAT3 positive feedback loop in human CRC development and progression. They demonstrated that miR-221/222 regulates NF-*κ*B and STAT3 signaling by directly binding to the *rela* coding region and stabilizing *rela* mRNA. Besides, miR-221/222 upregulates both RelA and STAT3 protein through binding to the 3′UTR of PDLIM2 as the E3 ligase for both RelA and STAT3.

**Figure 4:**
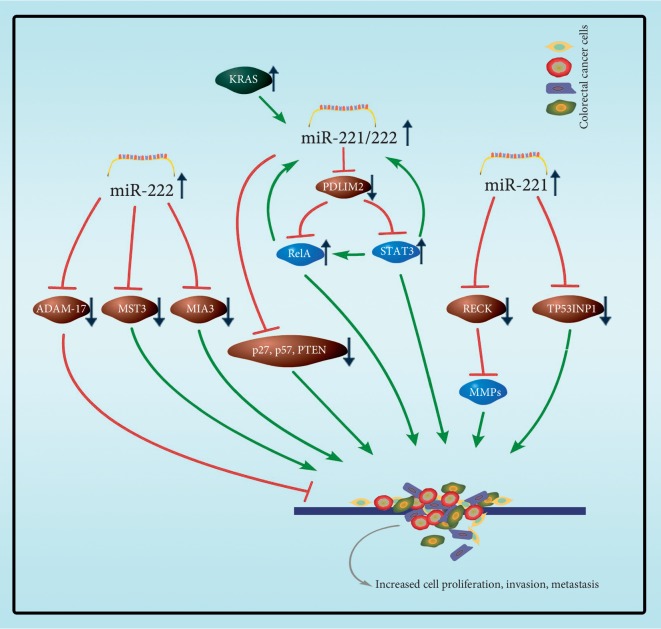
The main targets of miR-221/222 involved in colorectal cancer. miR-222 directly targets ADAM-17, which was downregulated in multidrug-resistant CRC cells and increase cancer cells' apoptosis. Meanwhile, miR-221/222 is an onco-miRNA in colorectal cancer cells. MST3 and MIA3 have been identified as a target for miR-222. RECK and TP53INP1 have been identified as a target for miR-221. p27/*CDKN1B*, p57/*CDKN1C*, and PTEN have been identified as a target for miR-221/222. The downregulation of these targets promotes colorectal cell proliferation, invasion, and metastasis.

The overwhelming cause of death from CRC is metastasis and reversion (Figure 4) [112, 113]. Qin and Luo [114] found miR-221 modulates CRC cell migration and invasion in vitro and in vivo, and they demonstrated that miR-221 could directly bind to reversion-inducing-cysteine-rich protein with Kazal motifs (RECK) 3′UTR and promotes metastasis in CRC. More reports demonstrated that mammalian STE20-like protein kinase 3 (MST3) may be the miR-222 target, and therefore, overexpression of miR-222 plays a critical role in regulating CRC cell migration and invasion [56]. Gao et al. [55] demonstrated that miR-222 enhances the migration and invasion in CRC cells by specifically targeting on the 3′UTR of melanoma inhibitory activity member 3 (MIA3). However, miR-222 may be a tumor suppressor miRNA in CRC [92]. Scientists investigated the role of ADAM-17 (a disintegrin and metalloprotease 17) as a novel multidrug resistance (MDR) mechanism in multidrug-resistant CRC and found that miR-222 was directly targeting on ADAM-17, which was downregulated in multidrug-resistant CRC cells and increased cancer cells' apoptosis.

### 3.3. Role of miR-221/222 in Cervical Cancer

Although a great deal of scientific research has been made in recent years, the mortality rates of gynecological cancers continue to rise [97, 115]. Early diagnosis and limited options of treatment for advanced gynecological cancers are the factors to their high mortality. However, the expression level of miRNAs in gynecological cancers is closely related to its disease diagnosis, clinic pathological features, and prognostic markers, while it has been found that miR-221/222, as a common ectopic miRNA, is almost at the abnormal expression level in gynecological cancer [116].

Sun et al. [67] observed that the expression of miR-222 in cervical cancer tissue was 2.48 times higher than that in normal tissues adjacent to cancer according to the samples of 18 patients. The upregulation of miR-222 was correlated with the deterioration of cervical cancer, and the analysis showed that p27/Kip1 and PTEN were negatively correlated with miR-222, which affected the proliferation and migration of Hela cells and SiHa cells, suggesting that miR-222 might be a new target for cervical cancer therapy. Wilting et al. [95] analyzed different expression profiles of miRNAs in the development of cervical cancer, and they found that the high expression of miR-221 was a specific marker of HPV positive cervical cancer. Cervical squamous cell carcinoma (CSCC), the most common histological subtype of cervical cancer, spreads principally by migrating into the lymphatics or by invading adjacent soft tissue. Wei et al. [116] demonstrated that there was a higher level of miR-221-3p, in human primary cervical cancer tissues derived from cervical cancer patients with or without lymph node metastasis, and it enhanced the malignancy of cervical cancer cells. The calculation and experiment showed that twist homolog 2 (TWIST2) targeted on the promoter region of miR-221-3p.

**Figure 5:**
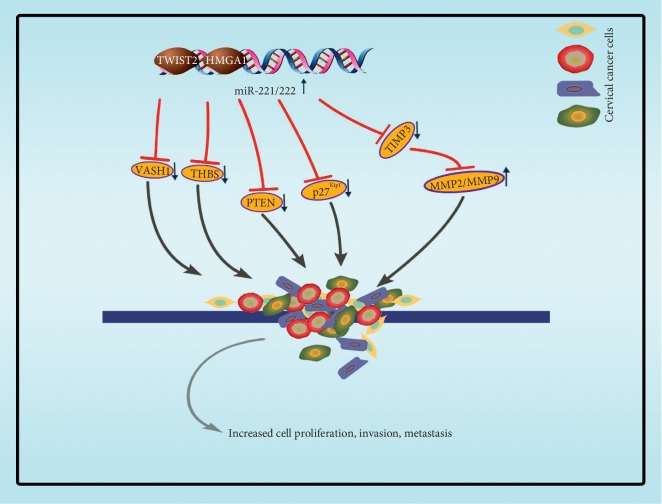
The main targets of miR-221/222 involved in cervical cancer. TWIST2 targets the promoter region of miR-221-3p and HMGA1 targets the promoter region of miR-221/222, both to enhance the expression of miR-221/222 in cervical cancer cells. P27/Kipq and PTEN have been identified as a target for miR-222. THBS2 and VASH1 have been identified as a target for miR-221. And miR-221/222-TIMP3-MMP2/MMP9 axis participates in the migration and invasion process.

As a consequence of the metastasis of cervical cancer lymphocytes, the transcription and expression of miR-221-3p were stimulated, and miR-221-3p targeted on the 3′UTR of twist homolog 2, thus accelerating the metastasis of cervical cancer lymphocytes. Some latest reports confirms that the miR-221-3p is characteristically enriched in and transferred by CSCC-secreted exosomes into human lymphatic endothelial cells (HLECs) to promote HLECs migration and tube formation in vitro and facilitate lymph angiogenesis and lymph node (LN) metastasis in vivo according to both gain-of-function and loss-of-function experiments. Furthermore, they identify vasohibin-1 (*VASH1*) as a novel direct target of miR-221-3p through bioinformatics target prediction and luciferase reporter assays [64]. High-mobility group AT-hook1 (HMGA1, formerly HMG-I/Y), an architectural transcription factor, participates in a number of tumor biological processes. Fu et al. [68] have shown that HMGA1 is highly expressed in CSCC tissue, which promotes the migration and invasion of cervical cancer cells and showed that the mechanism of HMGA1 cancer promotion is targeting the promoter region of miR-221/222 to enhance the expression of miR-221/222. Among them, miR-221/222 targeted on the 3′UTR of tissue inhibitor of metalloproteinases 3 (TIMP3), while TIMP3 downregulated and MMP2/MMP9 upregulated, and miR-221/222-TIMP3-MMP2/MMP9 axis participates in the migration and invasion process (Figure 5).

### 3.4. Role of miR-221/222 in Ovarian Cancer

Epithelial ovarian cancer (EOC) is the most common type of ovarian cancer, accounting for 90% of the total ovarian cancer, and the 5-year survival rate is relatively low, which is the main cause of death in gynecological malignant tumors [117, 118]. Therefore, early detection and chemosensitivity are essential to control disease development and reduce mortality. Some research found that the expression of miR-221 and miR-222 in human ovarian cancer tissues and cell lines reaches the highest levels of miRNA relative to immortalized ovarian surface epithelial cultures [119, 120]. Studies have shown that miR-222 is overexpressed in epithelial ovarian cancer cases and promotes cell proliferation through downregulation of p27^Kip1^ [71]. Shang et al. indicted that patients with positive expressions of human epidermal growth factor receptor 2 (HER2), signal transducer, and activator of transcription 3 (STAT3) and p-STAT3 or with negative expressions of suppressors of cytokine signaling 3 (SOCS3) had shorter survival time [72]. Moreover, Ying et al. indicate miR-222-3p was enriched in exosomes released from EOC cells and it could be transferred to macrophages. Overexpression of miR-222-3p in macrophages induced polarization of the M2 phenotype. TargetScould prediction and the luciferase assay proved that the 3′ UTR of *SOCS3* was targeted by miR-222-3p in these studies. Downregulation of SOCS3 correlated with an increased expression of *STAT3* activation and induced activation of JAK/STAT pathway, which increased M2 macrophages and promoted angiogenesis and lymphangiogenesis in tumor microenvironment, which accelerated progression of EOC [121].

More studies showed that the miR-221 was upregulated in 63 samples of ovarian cancer tissues, and miR-221 level was upregulated with larger tumor size, deeper tumor invasion, and higher FIGO stage. There was a negative correlation between the expression of apoptosis protease activator 1 (APAF1) protein and miR-221 in 5 of 63 ovarian cancer tissues and 6 cell lines, including A2780, OVCAR3, SKOV3, and 3AO5. The APAF1 gene was confirmed to be a direct target of miR-221, which induced the proliferation of ovarian cancer cells and hindered the apoptosis of ovarian cancer cells in vitro [69]. MiR-221 also targets on BCL-2 modifying factor (BMF) promoting cell proliferation in ovarian cancer cell line SKOV3 [70]. Amini-Farsani et al. demonstrated that, in human epithelial ovarian cancer cell lines A2780 S and A2780/CP, miR-221/222 was expressed at a higher level in A2780/CP cells. An in vitro cell viability assay showed that downregulation of miR-221/222 sensitized A2780/CP cells to cisplatin-induced cytotoxicity. Bioinformatics analysis and luciferase reporter assays proved that miR-221/222 was found to directly target on *PTEN*, and miR-221/222 induced cisplatin resistance by targeting *PTEN*-mediated PI3K/Akt pathway [73].

Like the abovementioned cancers, other studies have also found that the high expression of miR-222-3p and miR-221-3p has an anticancer effect. For example, high expression of miR-222-3p could inhibit the deterioration of cancer cells, and the patients with high expression seem owning higher survival rate and longer survival time. Fu et al. analyzed miR-222-3p expression with qRT-PCR in 74 EOC patients, and the results suggest the higher the mean expression level of miR-222-3p, the longer the median overall survival time of EOC. The expression of miR-222-3p in six ovarian cancer cell lines (Tara R182, SKOV3, SKOV3DDP, SKOV3IP, HO8910, and HO8910-PM) was detected with qRT-PCR analysis, and then, low level of miR-222-3p was found in SKOV3/DDP and Tara R182 cells with high cell growth rate. SKOV3 and HO8910-PM cells with low cell growth rate expressed high level of miR-222-3p. The overexpression of miR-222-3p in the SKOV3/DDP cell line demonstrated that the migration of the tumor cells was blocked. When miR-222-3p was overexpressed in six cell lines, it was found that miR-222-3p targets on AKT phosphorylation protein regulator G protein alpha inhibiting activity polypeptide 2 (GNAI2), which inhibited AKT phosphorylation to reduce the proliferation of ovarian cancer cells [91]. Recently, there are also some investigations showing that higher expression of miR-221-3p is associated with better overall survival in EOC patients. Wu et al. examined the expression level of miR-221-3p in three EOC cell lines (SKOV3, SKOV3-IP, and SKOV3-R), which showed lower expression level in SKOV3-IP and SKOV3-R but showed more proliferations and migrations.

**Figure 6:**
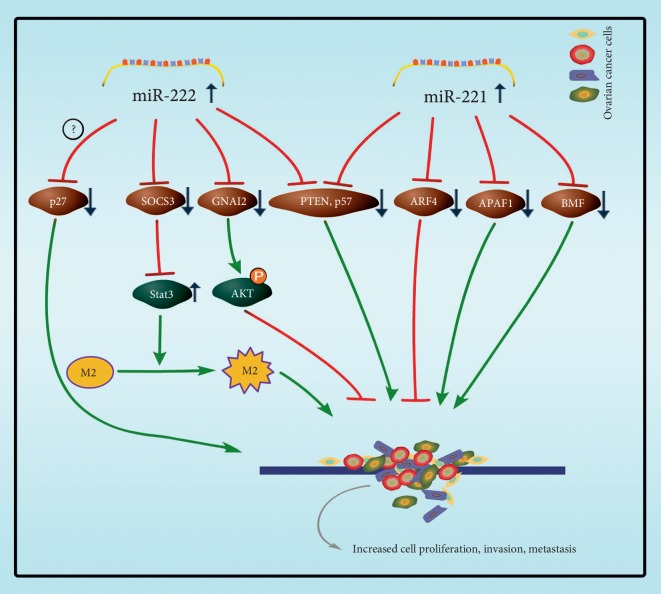
The main targets of miR-221/222 involved in ovarian cancer. Overexpression of miR-222 inhibits SOCS3 in progression of EOC, and downregulation of SOCS3 increased expression of STAT3 activation, inducing activation of JAK/STAT pathway, which increased M2 macrophages and promoted angiogenesis in tumor microenvironment. Upregulated miR-222 inhibits GNAI2 expression, which inhibited AKT phosphorylation to reduce the proliferation of ovarian cancer cells.

In vitro experiments indicated that miR-221-3p inhibited EOC cell proliferation and migration. By performing subsequent systematic molecular biological, bioinformatics analyses and luciferase reporter assay, scientists found and confirmed ADP-ribosylation factor (ARF) 4 is miR-221-3p′s targeting genes [90]. Interestingly, Wurz et al. [66] found that the expression of *CDKN1C* (p57) protein was negatively correlated with miR-221/222 in EOC, while the expression of *CDKN1B* (p27) protein was not correlated with miR-221/222 in EOC (Figure 6).

### 3.5. Role of miR-221/222 in Endometrial Carcinoma

Endometrial carcinoma (EC) is the fourth most common malignant tumor in women in developed countries and the most common cancer in female genital tract; however, the incidence of endometrial carcinoma has been moving to the young population due to obesity and lifestyle factors in China [122]. Estrogen is a classic etiological factor for endometrial tumorigenesis [123]. In endometrial carcinoma, deregulated ER*α* caused by genomic or epigenetic aberrations was a prevalent phenomenon, which reduced the expression of ER*α* and associated with extensive invasion and high-stage and poor prognosis [124, 125].

Liu et al. found miR-222-3p expression was much lower in ER*α*-positive than in ER*α*-negative endometrial carcinoma tissue samples, and the level of miR-222-3p expression was lower in tumors of lower grades and earlier stage. TargetScould and luciferase reporter assays revealed that ER*α* was a target of miR-222-3p. Therefore, miR-222-3p was overexpressed in ER*α*-negative endometrial carcinoma tumors and was associated with high grade, late stage, and nodal metastasis, and high level of miR-222-3p was a mechanism for raloxifene resistance in endometrial carcinoma therapy. Moreover, a significant increase in serum miR-222 levels of endometrial carcinoma patients was found by qRT-PCR miRNA analysis in 46 EC patients and 28 women without cancer history. It may be used as a proper marker for the diagnosis of endometrial carcinoma in future [126].

## 4. Conclusion

As summarized above, microRNA-221/222 (miR-221/222) is a noncoding microRNA which is widely distributed in eukaryotic organisms and deeply involved in the posttranscriptional regulation of gene expressions. It is important that the miR-221/222 expression level is closely related to tumor stage and prognosis. It may be used as a biomarker for the diagnosis of premalignant tumors [104] and provide a new target for tumor therapy [8–33, 35, 38, 51, 94], as a therapeutic tool for drug resistance or sensitivity to anticancer drugs [127].

However, more questions also need to be answered as translated into clinical strategies. Firstly, given the complex networks in which cancer cells are located, the roles of miR-221/222 in various cells' background as well as its target genes need more micromesh clarifications. For example, a laboratory announced that miR-222 promotes the proliferation of human non-small cell lung cancer cell line H460 [128]; in contrast, at almost the same time, Yamashita and colleagues [129] demonstrated that miR-221/222 promoted the growth of H460 cell lines while miR-221 inhibited the growth of the other four NSCLC cell lines. Another example is that the low expression of miR-221/222 inhibited the malignant proliferation of vascular smooth muscle cells [130] but also inhibited the growth of myocardial cells and the increase of muscle cell proliferation markers induced by exercise [131]. It is worth noting that the model miRNA analyzed and transfected in tumor samples may have isomiR properties, which may cause some of the results to be contradictory.

Secondly, with the deepening of the research, the expression level of miR-221 and miR-222 is not proportional to the development of tumor, while its functions are abundant. Therefore, the ratio of miR-221 and miR-222 expression level is expected to become a new research field [66].

Finally, it is expected that a single miRNA could act as a therapeutic inhibitor towards the cancer cellular pathway by regulating the different genes involved in the network. For example, miR-221/222 could inhibit the expression of ER-*α* and FOXO3A transcription factors, which results in the overall change of gene expression through the expression inhibition of ER-*α* and FOXO3A at posttranscriptional stage. The suppressed ER-*α* has the ability to negatively regulate miR-221/222, and the suppressed *FOXO3A* blocked the transcriptional activation of *p27* and *Bim* [63]. This shows that the regulation of individual miRNA may have great therapeutic potential towards various kinds of cancers. Back to the clinical strategy, for effectively suppressing onco-miRNA, locked nucleic acid- (LNA-) based oligonucleotides has been demonstrated to have great potential as inhibitors of small RNA targets [132].

## Figures and Tables

**Table 1:** Role of miR-221/222 in various cancers and their direct target genes.

Roles in cancer	Cancer types	miR-221/222	Target genes	References
Onco-miRNA	Glioblastoma	Both	*puma*	[31]
	Gastric cancer	miR-222	*reck*, *hipk2*	[32, 33]
		Both	*pten*	[34]
	Bladder cancer	miR-222	*ppp2r2a*	[35]
	Lung cancer	miR-222	*socs3*, *ppp2r2a*	[36, 37]
	Liver cancer	miR-221	*p27*, *p57*, *ddit4*, *bmf*, *hdac6*, *adipor1*, *erα*, *caspase*-*3*, *socs3*	[38–46]
		miR-222	*ppp2r2a*, *gnai3*, *bbc3*, *pbx3*	[47–50]
		Both	*pten*, *timp3*, *p27*	[29, 51]
	Colorectal cancer	miR-221	*p27*, *p57*, *pten*, *tp53inp1*, *reck*	[49, 52, 53]
		miR-222	*pten*, *mst3*, *mia3*	[54–56]
		Both	*p27*, *p57*, *pten*, *pdlim2*	[57, 58]
	Breast cancer	miR-221	*A20*	[59]
		Both	*erα*, *esr1*, *mybl1*, *cdkn1b*, *bim*, *cdkn1c*, *pten*, *timp3*, *ddit4*	[60–63]
	Cervical cancer	miR-221	*vash1*, *thbs*	[64, 66]
		miR-222	*p27*, *pten*	[67]
		Both	*timp3*	[68]
	Ovarian cancer	miR-221	*apaf1*, *bmf*,	[69, 70]
		miR-222	*p27*, *socs3*, *gnai2*	[71, 72]
		Both	*p57*, *pten*	[66, 73]
	Endometrial carcinoma	miR-222	*erα*	[74]
	Melanoma	Both	*p27*, *c*-*kit*	[75, 76]
	Pancreatic cancer	miR-222	*p27*	[77]
	Thyroid cancer	Both	*p27*, *pten*	[78–79]
	Multiple myeloma	miR-221	*p27kip1*	[80]
	Chronic lymphocytic leukemia	Both	*p27*	[81]
	Oral carcinoma	Both	*p27*	[82]
	Retinoblastoma	Both	*ap2α*	[83]
	Nasopharyngeal carcinoma	miR-222	*pten*	[84]
	Prostate carcinoma	Both	*p27*	[85]

Tumor suppressor miRNA	Prostate cancer	Both	*ecm29*	[61]
	Erythroleukemia	Both	*kit*	[86]
	Gastrointestinal stromal tumors	Both	*kit*	[87]
	Tongue squamous cell carcinoma	miR-222	*mmp1*, *sod2*	[88]
	Cholangiocarcinoma	miR-221	*pik3r1*	[89]
	Ovarian cancer	miR-221	*arf 4*	[90]
		miR-222	*gnai 2*	[91]
	Colorectal cancer	miR-222	*adam-17*	[92]
	Medulloblastoma	miR-221	*eif5a2*	[93]

## Data Availability

Some or all data, models, or code generated or used during the study are available from the corresponding author by request.
